# Extreme tooth enlargement in a new Late Cretaceous rhabdodontid dinosaur from Southern France

**DOI:** 10.1038/s41598-017-13160-2

**Published:** 2017-10-26

**Authors:** Pascal Godefroit, Géraldine Garcia, Bernard Gomez, Koen Stein, Aude Cincotta, Ulysse Lefèvre, Xavier Valentin

**Affiliations:** 1 Directorate ‘Earth and History of Life’, Royal Belgian Institute of Natural Sciences, 1000 Brussels, Belgium; 2 Université de Poitiers, IPHEP, UMR CNRS 7262, 86073 Poitiers, France; 3 Laboratoire de Géologie de Lyon: Terre, Planète, Environnement, UMR CNRS 5276, 69622 Villeurbanne, France; 4 Chemistry Department: Analytical, Environmental and Geo-chemistry, Vrije Universiteit Brussel, 1050 Brussels, Belgium; 5 Department of Geology, University of Namur, 5000 Namur, Belgium; 6 Department of Geology, Liège University, 4000 Liège, Belgium; 7 Palaios Association, 86300 Valdivienne, France

## Abstract

Rhabdodontidae is a successful clade of ornithopod dinosaurs, characteristic of Late Cretaceous continental faunas in Europe. A new rhabdodontid from the late Campanian, of southern France, *Matheronodon provincialis* gen. et sp. nov., is characterized by the extreme enlargement of both its maxillary and dentary teeth, correlated to a drastic reduction in the number of maxillary teeth (4 per generation in MMS/VBN-02-102). The interalveolar septa on the maxilla are alternately present or resorbed ventrally so as to be able to lodge such enlarged teeth. The rhabdodontid dentition and masticatory apparatus were adapted for producing a strict and powerful shearing action, resembling a pair of scissors. With their relatively simple dentition, contrasting with the sophisticated dental batteries in contemporary hadrosaurids, *Matheronodon* and other rhabdodontids are tentatively interpreted as specialized consumers of tough plant parts rich in sclerenchyma fibers, such as *Sabalites* and *Pandanites*.

## Introduction

Rhabdodontids are basal iguanodontian dinosaurs and characteristic elements of Late Cretaceous dinosaur faunas in Europe^1–4^. They have also been described in Early Cretaceous deposits of Spain^5^. Rhabdodontids are commonly represented, in late Campanian-early Maastrichtian dinosaur faunas of southern France, by two species of the genus *Rhabdodon*: *R*. *priscus* and *R*. *septimanicus* ^1,2^. Rhabdodontid disarticulated elements have recently been discovered at Velaux-La Bastide Neuve, Bouches-du-Rhône Department, southern France. This locality has yielded an abundant and diversified vertebrate fauna, including the titanosaurid sauropod *Atsinganosaurus velauciensis* ^6^, ankylosaurian remains, theropod teeth, an ontogenetic series of cranial and postcranial elements of the basal eusuchian crocodile *Allodaposuchus precedens* ^7^, pleurodiran and cryptodiran turtles, pterosaurs, hybodont shark teeth, and mawsoniid bones. Here we describe a new rhabdodontid dinosaur, *Matheronodon provincialis*, from Velaux-La Bastide Neuve, with a quite unusual dentition.

**Institutional Abbreviation**. MC, Musée de Cruzy, France; MMS/VBN, Musée du Moulin seigneurial/Velaux-La Bastide Neuve. The fossil material is labeled and housed in the municipality palaeontological and archeological structures of Velaux, is under the care of the research association Palaios, and is the property of the department DG 13.

Ornithischia Seeley, 1887

Ornithopoda Marsh, 1881

Iguanodontia Sereno, 1986

Rhabdodontidae Weishampel, Jianu, Csiki, and Norman, 2003

***Matheronodon provincialis*** gen. et sp. nov.

**Etymology**. *Matheron*: in honor of Philippe Matheron, who was the first to describe dinosaur remains in Provence; *odous* (Greek): tooth; *provincialis* (Latin): from Provence (southern France).

**Holotype**. MMS/VBN-02-102, a right maxilla; housed in the collections of the Musée du Moulin seigneurial (MMS, Velaux, France).

**Referred material.** MMS/VBN-93-34, MMS/VBN-09-149a, and MMS/VBN-09-150: maxillary teeth; MMS/VBN-12-22: maxillary tooth crown; MMS/VBN-02-11, MMS/VBN-09-43c, and MMS/VBN-12-A002: dentary teeth.

**Horizon and locality.** ‘Begudian’ (local stage) sandstones, late Campanian, Late Cretaceous^6,8^. Velaux-La Bastide Neuve, Aix-en-Provence Basin, Bouches-du-Rhône, southern France.

**Diagnosis.** Rhabdodontid ornithopod characterized by the following autapomorphies: enlargement of both maxillary and dentary teeth (up to 5 cm in mesio-distal length); reduction of maxillary tooth families (4 per generation in MMS/VBN-02-102); interalveolar septa on the maxilla alternately present or resorbed ventrally, so that one functional tooth is lodged in two paired alveoli; shortened rostral process on the maxilla; broad dorsal shelf along the rostral third of the maxilla; more than 25 vertical and parallel ridges on the labial side of the maxillary teeth.

## Description

**Figure 1:**
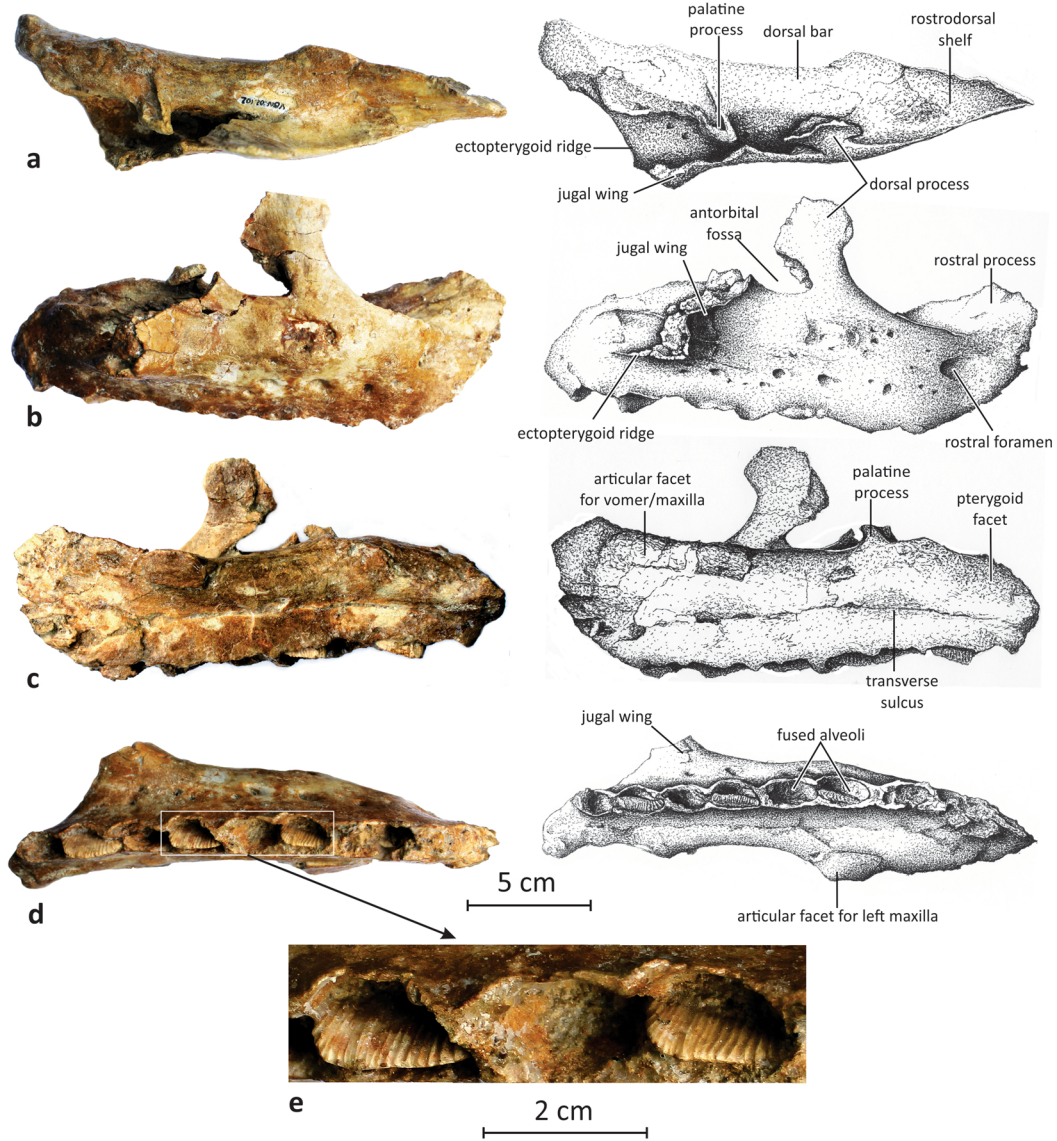
Right maxilla of *Matheronodon provincialis* gen. et sp. nov. (MMS/VBN-02–102; holotype) in dorsal (**a**), lateral (**b**), medial (**c**), and ventral (**d**) views. (**e**) Close-up of the second and third maxillary crowns.

**Figure 2:**
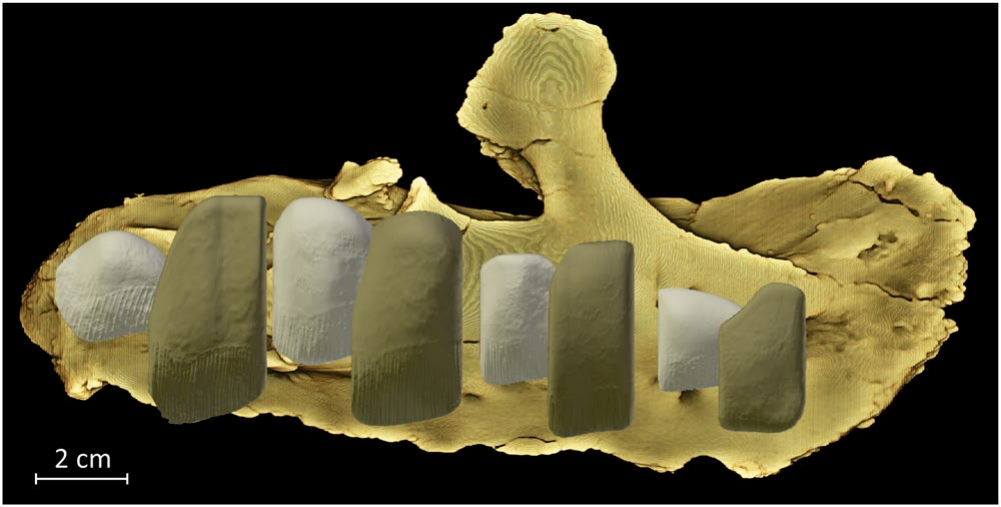
Reconstruction of the maxillary dentition of *Matheronodon provincialis* gen. et sp. nov. in lateral view from CT scans of MMS/VBN-02-102.

MMS/VBN-02-102 is a particularly robust right maxilla (rostrocaudal length of 22 cm and maximal height of 10 cm; Fig. 1). The rostral process is particularly short and oriented craniodorsally. In *Rhabdodon*, the rostral process is much more elongated, forming a horizontal styloid premaxillary process that is nearly as long as the body of the maxilla^9^; this styloid process is also well developed in *Zalmoxes* ^3^. The rostral border of MMS/VBN-02-102 is not eroded, so preservation cannot explain the shortening of its rostral process. In dorsal view, the rostral third of the maxilla is medially broadened to form a triangular and concave rostrodorsal shelf that likely supported the caudolateral (maxillary) process of the premaxilla; this shelf is absent in *Rhabdodon* ^9^ and *Zalmoxes* ^3^. The medial surface of the rostrodorsal shelf forms an elongated and flattened surface, likely marking the contact with the vomer and/or with the paired maxilla^3^ (Fig. 1c). Caudally to this surface, the dorsal half of the medial side of the maxilla is dorsoventrally convex, forming dorsal bar as also observed in *Rhabdodon* ^9^. At mid-length, the dorsal surface of the bar forms a prominent and laterally-oriented palatine process, with a deep groove along its mediocaudal side forming the articulation with the palatine (Fig. 1c). Caudally to the palatine process, the dorsomedial side of the dorsal bar is pitted and grooved by the articular surface for the pterygoid. The dorsal bar is separated from the flattened dentigerous ventral half by a continuous transverse sulcus (Fig. 1c), corresponding to the neurovascular groove that usually connects the alveolar foramina in ornithopods^10^. Caudally to the rostral process, the dorsolateral margin of the maxilla forms a prominent, dorsocaudally-inclined, dorsal process. The dorsal portion of this process is expanded along both its rostral and caudal edges; the caudal expansion is more important, forming a triangular caudodorsal wing that participates in the rostrodorsal margin of the elliptical antorbital fossa (Fig. 1b). The dorsal process is much more developed in *Matheronodon* than in *Zalmoxes* ^3^; in *Rhabdodon*, the dorsal process looks rostrocaudally narrower and is more vertically oriented, as observed in MC-QR9^9^. A large wing-like jugal process extends caudolaterally from the base of the dorsal process up to the caudal quarter of the bone (Fig. 1a,b). It is mediolaterally thin, separated from the main body of the maxilla by a wide and deep sulcus. Along its dorsal edge, a small triangular process marks the caudal end of the antorbital fenestra. A similar process is also present, though it is proportionally higher, in *Rhabdodon* ^9^. A short, but strong ectopterygoid ridge connects the caudal end of the jugal process to the main body of the maxilla (Fig. 1a,b). As in *Zalmoxes* ^3^, the lateral surface of the maxilla overhangs the tooth row and forms a large labial recess. A dozen foramina are more or less aligned along the dorsal part of the dentigerous portion of the maxilla (Fig. 1b) and likely transmitted neurovascular bundles from the maxillary division of the trigeminal nerve and the maxillary artery^3^. The rostralmost foramen, just in front of the rostral process, is the largest. Ventrally, the tooth row is relatively straight and composed of only eight alveoli, despite of the large size of this specimen. Up to 10 alveoli are present in much smaller *Zalmoxes* specimens^3^ and 11 in MC-QR9, of similar size as MMS/VBN-02-102, from the Campanian of Quarante (Hérault, southern France) and referred to as *Rhabododon* ^9^. The interalveolar septa are alternately present or nearly completely resorbed, so the base of the maxillary dental battery in fact contains four tooth positions, each of which is composed of two partially fused alveoli (Fig. 1d). Alveolar resorption has not been observed in *Rhabdodon* ^9^ and *Zalmoxes* ^3^. The functional maxillary teeth of MMS/VBN-02-102 are not preserved, but the crowns of the replacement teeth emerge from the alveoli. CT-scans of MMS/VBN-02-102 reveal the presence of two generations of replacement teeth (Fig. 2). The first generation emerges from the rostral half of each paired alveolus. The development of the maxillary teeth remained typically reptilian in pattern, progressing in wave-like fashion from the caudal part to the rostral part of the battery^11^. A second generation of replacement teeth is present in the caudal half of each paired alveolus. It can therefore be concluded that successive generations of teeth alternatively emerged from the rostral, and then from the caudal half of the paired fused alveoli. However, the tooth arrangement and replacement pattern in *Matheronodon* is not radically different from those in more advanced iguanodontians^11^. Because of the enlargement of the maxillary crowns (see below), teeth within each fused alveolus were largely imbricated, the rostral one covering the distal one in labial view (Fig. 2). Although they are only partially erupted and a large portion is consequently still embedded within the maxilla, more than 18 vertical ridges are visible along the labial surface of the second and third maxillary crowns (Fig. 1e), and the total number of labial ridges is consequently higher than 25 (see also Fig. 2).

**Figure 3:**
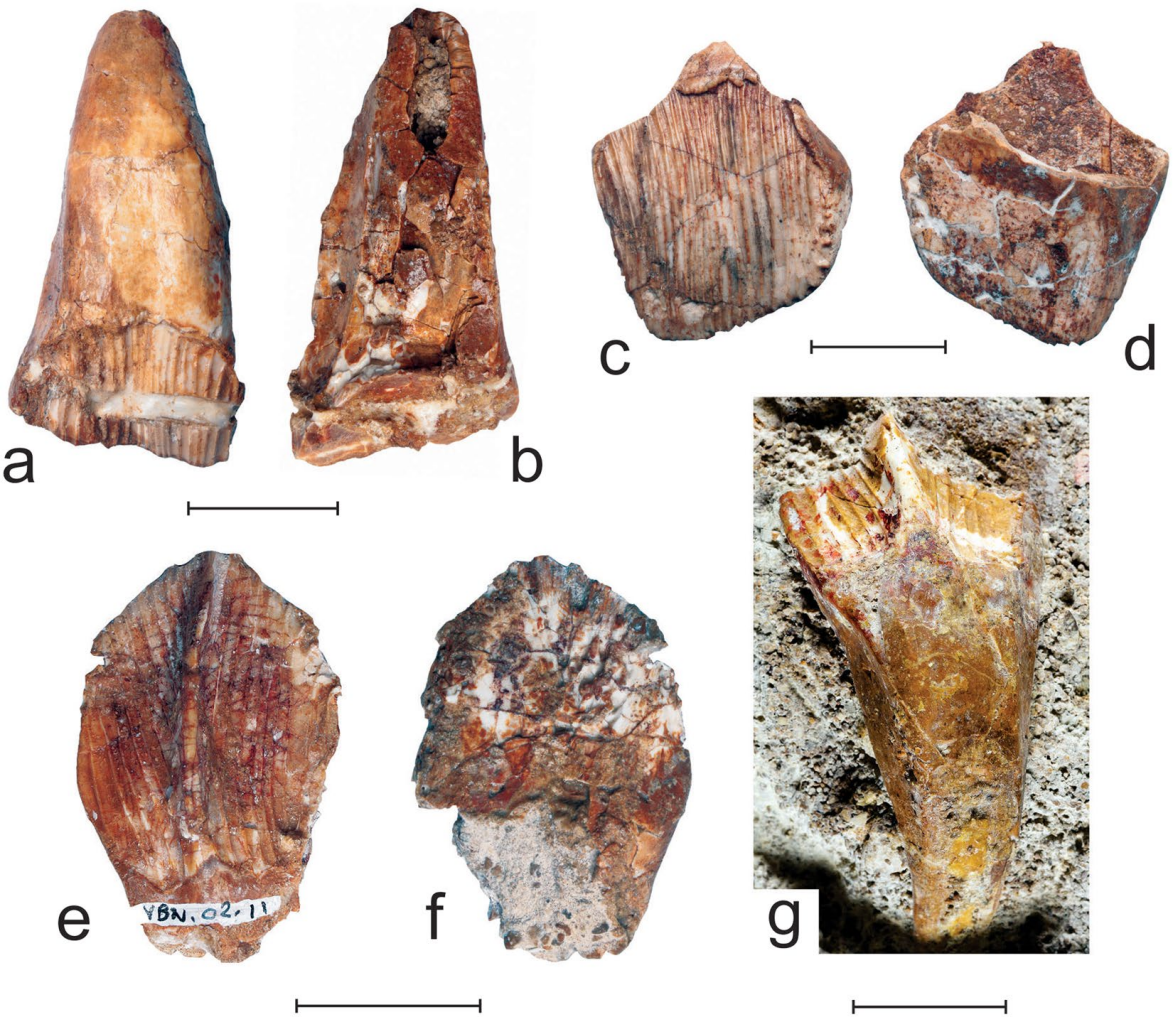
Isolated teeth of *Matheronodon provincialis* gen. et sp. nov. Left maxillary tooth (MMS/VBN- 09–149a) in labial (**a**) and lingual (**b**) views. Right maxillary crown (MMS/VBN-12-22) in labial (**c**) and lingual (**d**) views? Right dentary tooth (MMS/VBN-02-11) in lingual (**e**) and labial (**f**) views? Left dentary tooth (MMS/VBN-12-A002) in lingual view (**g**). Scale bars = 2 cm.

Isolated maxillary teeth (Fig. 3a–d) were found associated to MMS/VBN-02-102, but it cannot be ascertained that they belong to the same individual, as their size is rather heterogeneous and the fossils were obviously transported over a short distance^8^. However, all have the same morphology and also closely resemble those inside MMS/VBN-02-102, so they evidently belong to the same taxon. All are particularly high (around 6 cm in MMS/VBN-09-149) and mesiodistally broad (around 5 cm in MMS/VBN-09-150). Their crown is typically cleaver-shaped, as also observed in other rhabdodontids including *Zalmoxes* ^3^, *Mochlodon* ^4^, and *Rhabdodon* ^9^. In labial view, the crown is more elevated distally than mesially and the cutting apical edge is oblique and straight. Enamel covers the crown on all sides, but it is much thicker labially than lingually. As also observed in the holotype MMS/VBN-02-102, the labial surface of all recovered maxillary teeth is ornamented by more than 25 vertical and parallel ridges, all subequal in size. All the ridges reach the apical edge of the crown, forming tiny denticles, and then extend farther along the apical portion of the lingual side of the unworn crowns. Some of the ridges are bifurcated at the base of the labial side. The number of labial ridges is much higher in *Matheronodon* than in other rhabdodontids: a maximum of 19, 13, and 12 labial ridges can be observed in *Zalmoxes* ^3^, *Mochlodon* ^4^, and *Rhabdodon* ^9^, respectively. As already observed^3,9^, there is apparently no significant correlation between tooth width and number of ridges on the maxillary teeth in rhabdodontids. Basally, the labial enamel surface of the maxillary teeth of *Matheronodon* is bordered by a thin crenulated cingulum that curves apically along its distal margin. A single wear facet can be observed in MMS/VBN-09-149a, forming an angle of approximately 60° with the horizontal plane, as also observed in the most heavily worn teeth of *Zalmoxes* ^3^ and *Rhabdodon* ^9^. Scratches on the worn dentine surface are parallel and vertically oriented, as also observed in *Zalmoxes* ^3^ and *Mochlodon* ^9^. The root of the maxillary tooth is particularly robust, higher than the crown, and lingually curved. Well-developed replacement tooth grooves along both the mesial and distal sides of the roots indicate that functional and replacement teeth were closely imbricated.

Three large dentary teeth (Fig. 3e–g) were also found in Velaux-La Bastide Neuve, close to the holotype maxilla. As in other rhabdodontids, unworn dentary crowns have a leaf-shaped, lingual aspect, dominated by a prominent primary ridge, slightly distal to the midline of the crown. As in *Zalmoxes* ^3,12^, the thickly-enameled-lingual side of the crown is covered, on either side of the median ridge, by a dozen vertical subsidiary ridges. Subsidiary ridges are less numerous in *Rhabdodon* (up to 8 on either side of the primary ridge^9^) and *Mochlodon* (4–7 on either side^4^). The subsidiary ridges are slightly divergent and, as it is also the case for the maxillary teeth, they extend onto the apical part of the labial side of the crown, forming small denticles as they cross the apical edge. Unlike in *Mochlodon* ^4^, the subsidiary ridges reach the basal margin of the enameled surface of the lingual side of the crown. The enamel is much thinner on the labial side than on the lingual side of the crown. Because these dentary teeth were found in the same fossiliferous pocket as the holotype maxilla and are also characterized by more numerous subsidiary ridges than in *Rhabdodon* as it is also the case for the maxillary teeth, it is reasonable to refer them to *Mochlodon*.

## Tooth microstructure

**Figure 4:**
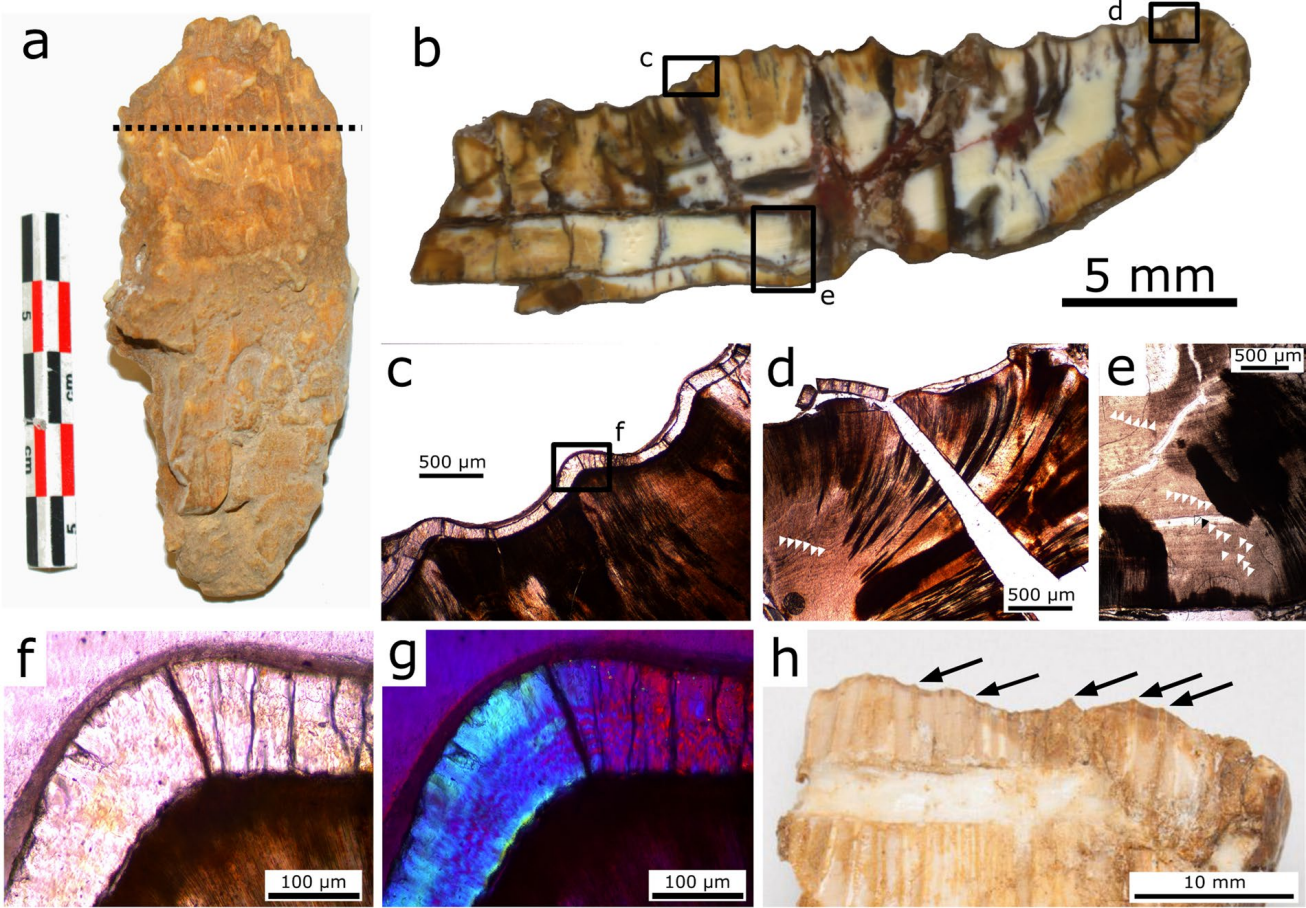
*Matheronodon* tooth histology. (**a**) Section plane in labiolingually compressed maxillary tooth MMS/VBN-93-34. (**b**) Incident light overview of a coronal section as indicated in (**a**,**c**) Transmitted light (plane polarized) microscopic view of boxed area in (**b**) showing enamel thickness variation on the ridges and grooves on the labial surface. (**d,e**) Transmitted light (plane polarized) microscopic views of boxed areas in (**b**) with details of the incremental lines in the dentine; Von Ebner lines are indicated by white arrows. (**f,g**) Transmitted light (plane polarized and cross polarized with lambda filter) microscopic view of boxed area in **c**, showing the enamel-dentine junction and wavy enamel Schmelzmuster. (**h**) Detail of the worn crown of MMS/VBN-09-149a (cf. Fig. 3a) showing an occlusal surface with a serrated (black arrows) slicing edge.

A coronal section in a maxillary tooth (Fig. 4) shows a jagged pattern of ridges and grooves on the labial surface (Fig. 4b,h), corresponding to the numerous vertical ridges, and a rather smooth surface on the lingual side (although small projections are also present). Measurements of enamel thickness (Supplementary Table S1) demonstrate that the ridges on the labial side of the crown have thicker enamel than the grooves in between. The enamel itself has a wavy enamel Schmelzmuster (Fig. 4f,g), similar to hadrosaurid dinosaurs^13,14^. Unlike in hadrosaurids^14^, the enamel is present around the entire crown of unworn teeth, but thinner on the lingual side of the sectioned specimen. Measurements on a well-preserved side of the tooth (Fig. 4c) show that *Matheronodon* has significantly thicker (Supplementary Table S1; independent t-test, N = 11, p < 0.001) enamel (avg. 179 µm) on its dental ridges, compared to the enamel on the central keel of *Edmontosaurus* teeth (avg. 155 µm, cf. also ‘*Anatosaurus*’^13^).

The dentine of the sectioned tooth (Fig. 4d,e) does not show any clear variation in the orientation of dentine tubules as in hadrosaurids^14^. We could also observe a high number of incremental Von Ebner lines (50 to 100 IVELs, Fig. 4e), indicating a long development time for a single tooth^15^. Unfortunately, the preservation state of the tooth did not allow a precise count of Von Ebner lines in the dentine.

## Discussion

Among basal iguanodontians, Rhabdodontidae (all iguanodontians more closely related to *Rhabdodon priscus* than to *Parasaurolophus walkeri* ^16^) form a clade mostly endemic to Europe during the Cretaceous. The following dental characters, regarded as unambiguous synapomorphies for Rhabdodontidae^3–5^ can be observed in *Matheronodon*: dentary teeth with multiple (more than 10) evenly-spaced accessory ridges around the primary ridge, and maxillary teeth with multiple ridges of similar size and devoid of a primary ridge. Besides *Matherodon provincialis*, three rhabdodontid genera and six species have currently been named: *Rhabdodon priscus* from the Campanian-Maastrichtian of France and Spain^1^, *R*. *septimanicus* from the Campanian-Maastrichtian of France^2^, *Zalmoxes robustus* and *Z*. *shqiperorum* from the Maastrichtian of Romania^3,17^, *Mochlodon suessi* from the Campanian of Austria^18^, and *Mochlodon vorosi*, from the Santonian of Hungary^4^.

The maxilla MMS/VBN-02-102 and the teeth that were found in the same fossiliferous pocket at Velaux-La Bastide Neuve are quite different from those described in *Rhabdodon*, also from late Campanian-early Maastrichtian deposits from Southern France, and in other rhabdodontids, so they clearly belong to a new taxon: the rostral process is short and rostrodorsally oriented, although it is much more elongated, horizontal and styloid in *Rhabdodon*; the dorsal shelf is much broader on the rostral half of the maxilla; the dorsal process is wider and caudally inclined in MMS/VBN-02-102; alveolar resorption has not been observed in *Rhabdodon* so far; the number of labial ridges is much higher on the maxillary teeth in *Matheronodon* (>25) than in *Rhabdodon* (<12); and the number of subsidiary ridges is also higher on the dentary teeth in *Matheronodon* (a dozen or more on either side of the primary ridge) than in *Rhabdodon* (up to 8 on either side of the primary ridge).

The sophistication of their feeding apparatus has long been identified as a key element in the evolutionary success and diversification of ornithopods^19,20^. Basal, non-iguanodontian ornithopods are characterized by relatively low tooth count (14–17 positions), low, labiolingually-compressed tooth crowns that are bulbous to sub-triangular in shape, and ornamented by numerous apicobasal ridges^21–23^. The marginal denticles are relatively large and triangular^20^. More advanced iguanodontians, such as *Camptosaurus dispar* ^24^, *Iguanodon bernissartensis* ^25^, *Mantellisaurus atherfieldensis* ^26^, and *Ouranosaurus nigeriensis* ^27^, are characterized by a higher tooth count (around 20 positions), proportionally higher, shield-like tooth crowns, a reduced number of apicobasal ridges, and proportionally reduced marginal denticles. The evolutionary explosion of hadrosauroid iguanodontians during the Late Cretaceous was accompanied by the development of true dental batteries, consisting of up to 60 closely packed tooth families. In advanced hadrosaurids, each tooth family is made of three up to seven successional teeth. Tooth miniaturization is accompanied in hadrosaurids by proportionally higher tooth crowns and by a further reduction of ridges to a single median carina (one or two faint subsidiary ridges are occasionally present) and of the marginal denticles^11^. Enamel restriction to the sides of crowns promoted self-sharpening^14^. In most hadrosaurids, up to three successional teeth from each tooth row participate in the occlusal surface, forming labiolingually wide pavements across the entire chewing area. This morphological complexity is coupled, in hadrosaurids, with a histological complexity and the development of six dental tissues instead of two in basal genasaurans, for example^14^. Hadrosaurids therefore developed a complex, dual-function slicing-grinding system presumably for the consumption of moderately tough, cellulose-rich plant diet^14^.

The rhabdodontid dentition evolved in an opposite way: unlike hadrosaurids, they developed a reduced number of mesiodistally wide, but labiolingually thin teeth^4^. Extreme tooth enlargement can be observed in *Matheronodon*, in which the number of maxillary tooth rows is reduced to eight. The high-angled wear surface has a particularly sharp, chisel-like cutting edge. Scratches on the worn dentine surface are usually well marked, parallel and vertically oriented^3,4^. Moreover, the rhabdodontid quadrate was proportionally low and massive^3,9^, so that the jaw joint was likely not offset too far from the level of the tooth row. The rhabdodontid skull was likely triangular in dorsal view: the premaxillae and predentary of *Zalmoxes* are narrow, whereas the maxillae diverge strongly posteriorly, and the quadrates are laterally splayed^3^. Intracranial mobility was obviously much more limited than in hadrosaurids. In rhabdodontids, the robust jaw, strong and elevated coronoid process and large jaw adductor muscle chamber suggest that jaw closure was powerful and that the motion of lower jaw was limited by the complex jaw joint and tight predentary-dentary suture^3^. All these characters suggest that the rhabdodontid dentition and masticatory apparatus were adapted for producing a strict and powerful slicing action, resembling a pair of scissors.

The peculiar *Matheronodon* tooth microstructure also reflects specialization for slicing. As soon as a new tooth erupted, the ridges along the wear-resistant thicker enameled side of the crown formed a self-sharpening serrated and jagged slicing edge (Fig. 4h). Precise measurement of durability would require detailed tribological measurements^14,28^, but this is beyond the scope of the current paper. Due to the high tooth development time and large size of the teeth, the tooth replacement rate of *Matheronodon* was probably not as high as in hadrosaurids or ceratopsians^14,28^.

From a biomechanical point of view, enlarged blade-like teeth, as exemplified by *Matheronodon*, and also by the mammalian carnassial teeth, are best adapted for fracturing tough (= resistant to crack propagation) foodstuffs^28–31^. Hadrosaurids, on the contrary, developed a much more complex dentition forming a dual-function shearing-crushing system, suggestive of a broader dietary range, including leaves, fruits, seeds, and twigs^32–34^; subtle variations in their tooth morphology would have allowed for diversification into more specialized ecological niches^14,31^. In any case, the important morphological differences in their dentitions likely lead to niche portioning between rhabdodontids and hadrosaurids in Europe by the Late Cretaceous time.

Besides *Matheronodon*, the Velaux-La Bastide Neuve locality has yielded other herbivorous dinosaur remains, including ankylosaurid elements, and abundant material of the titanosaurid sauropod *Atsinganosaurus velauciensis* ^6^. We have not found any trace of hadrosauroids yet. Previous works based on sites in southern France have concluded that an important faunal replacement related to an important late Maastrichtian marine regression occurred in southern Europe during the Late Cretaceous: a Campanian - early Maastrichtian fauna dominated by titanosaurids and rhabdodontids was replaced by a late Maastrichtian assemblage dominated by hadrosaurids^35^. However, the presence of hadrosauroids, titanosaurids and rhabdodontids in the late Maastrichtian assemblage of Vitrolles-La-Plaine suggests that these animals were still living together in southern France during the latest Cretaceous, but maybe in different environments. Their co-occurrence in the allochthonous assemblage of Vitrolles-La-Plaine can easily be explained by taphonomic processes (hydraulic transport) that concentrated, in the same level, bones of animals living in different places^36^.

Ceratopsians are particularly rare in Late Cretaceous deposits of Europe. Scarce remains have been described from the Coniacian-Santonian of Belgium^37^ and from the Campanian of Sweden^38^. The coronosaurian neoceratopsian *Ajkaceratops kozmai* was discovered in the Santonian Csehbánya Fm of Iharkút in Hungary^39^, which has also yielded the rhabdodontid *Mochlodon vorosi* ^4^. In contrast, ceratopsians are abundant in Late Cretaceous deposits of Asia and North America, whereas rhabdodontids have not been reported from these areas. Ceratopsian dentitions produced a strict shearing action^31^, as also hypothesized for rhabdodontids in the present paper. So, both the medium-sized ceratopsians and the rhabdodontids potentially fed on similar tough, woody or fibrous vegetation.

What *Matheronodon* and *Rhabdodon* fed on will probably remain unknown, but this issue can be discussed based on some plant fossil assemblages known during the Campanian-Maastrichtian of Europe (see Supplementary Information for more detailed information on the composition of those assemblages): the Grünbach Formation in Austria^40–42^; the Haţeg and Rusca Montană basins in western Romania^43^, which have also yielded abundant remains of the rhabdodontid *Zalmoxes*; the Áger, Vallcebre Coll de Nargό and Tremp basins in the southern Spanish Pyrenees^44–60^; Lo Hueco in the south-western Iberian Ranges^61,62^, which has also yielded fossils of rhabdodontid and titanosaurid dinosaurs; and Fuveau Basin, Etang de Berre, and Sainte-Baume Massif in southeastern France^63–65^. In Europe, plant assemblages strongly vary in richness and abundance depending on the localities, but overall plant megafossils are marked by the replacement of the conifer *Geinitzia* by *Cunninghamites* and the spread of the monocots *Sabalites* and *Pandanites*, while plant microfossils are characterized by the abundance of fern spores and Normapolles-group eudicot pollen grains.

Tough woody tissue exists in diverse vascular plant groups, such as gymnosperms and most angiosperms except monocots, and is found in leaves, stems, and roots. It is made of secondary xylem, commonly called wood, which consists of water and mineral conductive cells, called tracheids and vessels, parenchyma cells and facultative sclerenchyma fibers. Sclerenchyma also occur out of secondary xylem, and are particularly abundant in some large leaves such as in some palms; in leaves and stems of many herbaceous and tree plants, sclerenchyma serves the function of support when wood is absent. The relative abundance of secondary xylem *vs* sclerenchyma fibers in eaten plant parts such as leaves and stems may cause large herbivores to adapt their diet and tooth morphology. Soft eudicot leaves with few or no sclerenchyma fibers can be easily crushed with a pestle in a mortar. Campanian-Maastrichtian conifer leafy stems, such as *Geinitzia* and *Cunninghamites*, and eudicot leaves and small stems may be easily and quickly beat into pulp. So they do not only require powerful blade-like teeth. In contrast, many monocot leaves in particular in palms such as the Campanian-Maastrichtian *Sabalites longirhachis* and *Pandanites* spp. contain much sclerenchyma fibers along the parallel veins to stiffen the large leaf laminas and petioles. Such fibers must be cut into small fragments with blade-like teeth prior to be swallowed.

It might therefore be cautiously hypothesized that rhabdodontids were adapted to preferentially feed upon tough plant parts rich in sclerenchyma fibers such as *Sabalites* and *Pandanites*. In contrast, conifers potentially constituted an important part in the diet of North American hadrosaurids. The supposed gut content of an *Edmontosaurus* “mummy” mainly consists of conifer needles and branches, together with deciduous angiosperm foliages and numerous small seeds or fruits^32,66^. Coprolites tentatively referred to *Maiasaura* primarily contain conifer stem fragments^66^. Nevertheless, there is no indication of drastic changes in the character or composition of terrestrial vegetation between the early and late Maastrichtian that could easily explain the sudden abundance of hadrosaurids and the purported contemporary decline of rhabdodontids in western Europe. Correlating faunistic and floristic remains highly speculative in the current state of our knowledge.

## Methods

Computed tomography (CT) of the maxilla was done at the veterinary faculty of the University of Liege. A 16 multi-slice CT scan was used (Siemens, Somatom 16, Erlangen, Germany) with the following parameters; 120 kV and 46 mA under a Dental 0.75 H60s protocol. The slices of 0.5 mm in depth were digitally reconstructed using Amira 5.3.3 and Drishti v2.6.3.

For investigating tooth microstructure, we sectioned a slightly crushed and isolated maxillary tooth of *Matheronodon*. The specimen was embedded in epoxy resine (Araldite 2020) and sectioned and ground to a thickness of ~50 µm using standard lapidary methods.

### Nomenclatural Act

This published work and the nomenclatural acts it contains have been registered in ZooBank, the proposed online registration system for the International Code of Zoological Nomenclature. The ZooBank life science identifiers can be resolved and the associated information viewed by appending the life science identifiers to the prefix http://zoobank.org/. The life science identifiers for this publication is 1AD18D4B-7063–43F7-A55E-19463D066D78; for *Matheronodon*: FF4C534D-206A-4659–8592–9154ABF4A8B5, and *M. provincialis*: A6D62068–15A5–4ADE-83DD-74F0ED529BAB.

## Electronic supplementary material

Supplementary Information
