# Supplementary material for: Extreme tooth enlargement in a new Late Cretaceous rhabdodontid dinosaur from Southern France
Source: Sci Rep. 2017 Oct 26;7:13098. doi: 10.1038/s41598-017-13160-2 (PMC5658417; doi:10.1038/s41598-017-13160-2)
Supplement: Supplementary file 1 — Supplementary Information [file 41598_2017_13160_MOESM1_ESM.doc]

**Supplementary Information for:**

**Extreme tooth enlargement in a new Late Cretaceous rhabdodontid dinosaur from Southern France**

**Pascal Godefroit^1^, Géraldine Garcia^2^, Bernard Gomez^3^, Koen Stein^4^, Aude Cincotta^1,5^, Ulysse Lefèvre^1,6^, Xavier Valentin^2,7^**

^1^Directorate ‘Earth and History of Life’, Royal Belgian Institute of Natural Sciences, 1000 Brussels, Belgium. ^2^Université de Poitiers, IPHEP, UMR CNRS 7262, 86073 Poitiers, France. ^3^Laboratoire de Géologie de Lyon: Terre, Planète, Environnement, UMR CNRS 5276, 69622 Villeurbanne, France. ^4^Chemistry Department: Analytical, Environmental and Geo-chemistry, Vrije Universiteit Brussel, 1050 Brussels, Belgium. ^5^Department of Geology, University of Namur, 5000 Namur, Belgium. ^6^Department of Geology, Liège University, 4000 Liège, Belgium. ^7^Palaios Association, 86300 Valdivienne, France. Correspondence and requests for materials should be addressed to P.G. (email: [Pascal.Godefroit@naturalsciences.be](mailto:Pascal.Godefroit@naturalsciences.be))

| **Supplementary Table 1.** **Compared enamel thickness in *Matheronodon* (MMS/VBN-93-34; maxillary tooth) and *Edmontosaurus*^15^.**  **Enamel thickness (µm)** | | | |
| --- | --- | --- | --- |
| ***Matherondon* (MMS/VBN-93-34)** | | ***Edmontosaurus*^15^** | |
| concave surface | keel | concave surface | keel |
| 96,599 | 179,05 | 115,896 | 160,983 |
| 105,788 | 178,936 | 100,565 | 143,603 |
| 105,73 | 174,382 | 113,832 | 149,003 |
| 109,322 | 172,618 | 113,832 | 138,848 |
| 109,802 | 180,893 | 103,66 | 175,89 |
| 102,704 | 172,496 | 113,832 | 166,103 |
| 88,84 | 173,437 | 120,901 | 129,918 |
| 93,999 | 178,201 | 108,473 | 159,371 |
| 91,969 | 185,822 | 105,534 | 159,943 |
| 98 | 189,29 | 118,227 | 166,651 |
| 89,603 | 180,508 | 105,149 | 150,534 |
| 95,919 |  | 89,404 |  |
| 94,46 |  | 95,546 |  |
| 96,302 |  | 111,879 |  |
| 89,603 |  | 99,045 |  |
|  |  | 105,149 |  |
|  |  | 114,734 |  |

**Palaeobotanical assemblages from various campano-Maastrichtian deposits in Europe**

The early Campanian mire flora of the Grünbach Formation in Austria yielded 53 plant megafossils including ferns (11 taxa), horsetails (1 taxon), cycads (1 taxon), conifers (4 taxa), monocots (6 taxa), and eudicots (30 taxa)^41^. Spores and pollen of mosses, lycopods, ferns, gymnosperms, and angiosperm monocots and eudicots were also identified in this formation; the assemblage is dominated by *Normapolles*-group eudicot pollen grains.

The Maastrichian of the Haţeg and Rusca Montană basins in western Romania, which have also yielded abundant remains of the rhabdodontids *Zalmoxes*, shows distinct assemblages. The Haţeg plant megafossils consist of ferns (2 taxa), monocots (1 taxon) and eudicots (2 taxa), while the sporomorph assemblage contains mainly fern spores and *Normapolles* eudicot pollen grains^44^.The Rusca Montană flora includes horsetails (1 taxon), ferns (6 taxa), conifers (1 taxon), monocots (3 taxa) and eudicots (11 taxa)^44^.

Plant meso- and megafossils have been reported from the Maastrichtian of the Vallcebre^45-49^and Tremp^50-52^ basins, southern Pyrenees, Spain. They contain gymnosperms, monocots and eudicots. Plant microfossils from the Maastrichtian of Pyrenees have been studied in the Áger, Vallcebre, Coll de Nargό and Tremp basins^53-61^ and are particularly diversified, including freshwater green algae, hornworts, spike mosses, ferns, cycads, ginkgoes, and monocots and eudicots. In most localities fern spores are far more abundant than gymnosperm and angiosperm pollen grains.

The Campanian-Maastrichtian site of Lo Hueco (Cuenca, Spain), has recently yielded abundant and diversified fossils, including plant micro- (sporomorphs), meso- and mega-fossils, besides rhabdodontid and titanosaurid dinosaurs^62^. Plant megafossils consist of ferns, conifers and angiosperms, including a probable aquatic form. Some fragments of leaves showing parallel venation pattern suggest the occurrence of monocots. The angiosperm pollen accounts for around 80% of the sporomorph assemblage and is represented by more than 20 taxa^62-63^.

In France, the lower Campanian of Fuveau basin, of Etang de Berre and of Sainte-Baume massif, southeastern France yielded a rich megaflora collected in the late nineteenth century. It includes ferns (2 taxa), conifers (2 taxa), and angiosperm lotus (1 taxon), monocots (7-8 taxa) and eudicots (6 taxa)^64-65^. The palynoflora of the Campanian-Maastrichtian locality of Tercis-les-Bains (SW France) is largely dominated by angiosperms^66^.

Overall, the Campanian-Maastrichtian plant megafossil assemblages in Europe are marked by the replacement of the conifer *Geinitzia* by *Cunninghamites*^41^ and the spread of the monocots *Sabalites* and *Pandanites*, while microfossil assemblages are characterized by the abundance of fern spores and *Normapolles*-group eudicot pollen grains.
